# Establishing Classifiers With Clinical Laboratory Indicators to Distinguish COVID-19 From Community-Acquired Pneumonia: Retrospective Cohort Study

**DOI:** 10.2196/23390

**Published:** 2021-02-22

**Authors:** Wanfa Dai, Pei-Feng Ke, Zhen-Zhen Li, Qi-Zhen Zhuang, Wei Huang, Yi Wang, Yujuan Xiong, Xian-Zhang Huang

**Affiliations:** 1 Department of Respiration Gong An County People’s Hospital Jingzhou China; 2 Department of Laboratory Medicine The Second Affiliated Hospital Guangzhou University of Chinese Medicine Guangzhou China; 3 Guangdong Provincial Key Laboratory of Research on Emergency in Traditional Chinese Medicine Guangzhou China; 4 Second Clinical Medical College Guangzhou University of Chinese Medicine Guangzhou China

**Keywords:** COVID-19, clinical laboratory indicators, community-acquired pneumonia, classifier, classification algorithm

## Abstract

**Background:**

The initial symptoms of patients with COVID-19 are very much like those of patients with community-acquired pneumonia (CAP); it is difficult to distinguish COVID-19 from CAP with clinical symptoms and imaging examination.

**Objective:**

The objective of our study was to construct an effective model for the early identification of COVID-19 that would also distinguish it from CAP.

**Methods:**

The clinical laboratory indicators (CLIs) of 61 COVID-19 patients and 60 CAP patients were analyzed retrospectively. Random combinations of various CLIs (ie, CLI combinations) were utilized to establish COVID-19 versus CAP classifiers with machine learning algorithms, including random forest classifier (RFC), logistic regression classifier, and gradient boosting classifier (GBC). The performance of the classifiers was assessed by calculating the area under the receiver operating characteristic curve (AUROC) and recall rate in COVID-19 prediction using the test data set.

**Results:**

The classifiers that were constructed with three algorithms from 43 CLI combinations showed high performance (recall rate >0.9 and AUROC >0.85) in COVID-19 prediction for the test data set. Among the high-performance classifiers, several CLIs showed a high usage rate; these included procalcitonin (PCT), mean corpuscular hemoglobin concentration (MCHC), uric acid, albumin, albumin to globulin ratio (AGR), neutrophil count, red blood cell (RBC) count, monocyte count, basophil count, and white blood cell (WBC) count. They also had high feature importance except for basophil count. The feature combination (FC) of PCT, AGR, uric acid, WBC count, neutrophil count, basophil count, RBC count, and MCHC was the representative one among the nine FCs used to construct the classifiers with an AUROC equal to 1.0 when using the RFC or GBC algorithms. Replacing any CLI in these FCs would lead to a significant reduction in the performance of the classifiers that were built with them.

**Conclusions:**

The classifiers constructed with only a few specific CLIs could efficiently distinguish COVID-19 from CAP, which could help clinicians perform early isolation and centralized management of COVID-19 patients.

## Introduction

COVID-19 caused by SARS-CoV-2 infection, which was discovered in early December 2019, has become a global pandemic. As of August 3, 2020, COVID-19 has become widespread in 215 countries, areas, or territories worldwide; it has caused infection in more than 17.9 million people and has resulted in the deaths of more than 686,000 people [1]. The World Health Organization has stated that the spread of COVID-19 may be impeded by early detection, isolation, and the implementation of a robust health care system [2,3]. Nevertheless, the published data indicate that the initial symptoms of COVID-19 in patients are very similar to those in patients with the common cold or influenza. COVID-19 patients exhibit different clinical symptoms, and some of them do not have any symptoms [4-7]. sars-cov-2 infection has a long incubation period, with a median incubation period of 5 to 7 days, which is the chief risk factor for community infection [6,8]. Community-acquired pneumonia (CAP) and COVID-19 have similar clinical and imaging features, but their treatment and infectivity are very dissimilar. Distinguishing COVID-19 from CAP is very important to prevent the spread of COVID-19 and to provide specific treatment.

Some characteristic spectra demonstrated by clinical laboratory indicators (CLIs) of COVID-19 patients have been utilized as auxiliary clues for diagnosis [9]. Previous studies have demonstrated that increased procalcitonin (PCT), lymphocytopenia, and thrombin activation can all be utilized as auxiliary diagnostic indicators of COVID-19 and poor prognostic factors [9-11]. However, they are also correlated with CAP [12-15]. Thus, in accordance with the changes in these indicators, it is impossible to differentiate COVID-19 from CAP. The changes in the neutrophil to lymphocyte ratio, the peak platelet to lymphocyte ratio, lactate dehydrogenase (LDH), C-reactive protein (CRP), and interleukin-6 (IL-6) are considered to be associated with the progression and prognosis of COVID-19 [9], but using the information from the CLIs to give clinicians correct guidance is still a great challenge.

Classifiers established by machine learning (ML) algorithms based on various clinical features, biomarkers, and CLIs are increasingly widely utilized in disease diagnosis and risk prediction [16]. During the COVID-19 pandemic, ML was also widely used to predict, classify, assess, track, and control the spread of SARS-CoV-2 [17,18]. ML can improve diagnostic performance compared with hand-selected biomarkers by selecting relevant biomarkers and more consistently capturing both their relative importance to prediction and their interactions among one another [19]. In this study, we used CLIs to build classifiers with different ML algorithms to distinguish COVID-19 patients from CAP patients; we found that only the feature combinations (FCs) with many specific CLIs rather than the FCs with the most significantly differential CLIs between the two groups could build high-performance classifiers (HPCs).

## Methods

### Collection of Patients’ Electronic Medical Record Data

The electronic medical records of patients who were admitted to Gong An County People’s Hospital, China, and diagnosed with COVID-19 or CAP from December 2019 to March 2020 were retrieved. The information regarding each patient’s age, sex, clinical symptoms upon admission, medical history, epidemiological history, computed tomography (CT) imaging features, and CLIs were sorted out for retrospective analysis. Only the laboratory test results during admission were included. It was specified that all patients’ data were to be kept confidential, and this data were only to be utilized for comprehensive analysis. No personal information about any patient was mentioned in the paper. This study was approved by the ethics committees from the Guangdong Provincial Hospital of Chinese Medicine (approval No. ZE2020-049-01) with a waiver of informed consent due to the retrospective nature of the study.

### Data Description

Diagnosis and clinical classification of COVID-19 were performed according to the *Chinese Clinical Guidance for COVID-19 Pneumonia Diagnosis and Treatment (7th edition)* [20]. A total of 61 patients with COVID-19 and 60 patients with CAP were enrolled according to the discharge diagnosis on their electronic medical records. There were 3 mild, 47 common, 6 severe, and 5 critical types, which were categorized into two groups for further analysis as follows: COVID19-COM (3 mild and 47 common types) and COVID19-SV (6 severe and 5 critical types). They were matched by age and sex and did not significantly differ in terms of medical history. The main clinical symptoms between CAP and COVID-19 groups were not significantly different.

### Primary Analysis

The descriptive analysis of all CLIs was performed between groups or subgroups. Between-group or between-subgroup differences were tested using the *statsmodels* module from Python (Python Software Foundation) [21]. The Student *t* test was performed when the distribution of the variables conformed to the normal distribution; otherwise, the Mann-Whitney *U* test was used. The chi-square test was used to detect differences in baseline data between two groups or subgroups. A value of *P*<.05 was considered to be significant.

### Feature Selection and Data Preprocessing

The CLIs with a missing value ratio greater than 20% were excluded. Only the CLIs with a significant difference between the two groups were selected and used to generate 1,807,780 nonrepetitive random FCs, consisting of one to eight CLIs, by using the *combinations* iterator in the *itertools* module from Python [22]. Next, an FC was selected from the FC list one by one to form a new data sheet with the dependent variable (ie, disease type), and 1,807,780 new data sheets were eventually formed. For each new data sheet, the rows with missing values were removed. The remaining rows were then divided into *training_dataset* and *test_dataset* using scikit-learn, version 0.23.1 (*train_test_split* function with *test_size* = 0.25, *random_state* = 0). The training data set was used to build the classifier, and the test data set was used to assess the performance. The feature values were standardized using the *StandardScaler* function in the scikit-learn module before constructing the logistic regression (LR) classifier.

### Construction of Classifiers With ML Algorithms in the Scikit-Learn Module

Scikit-learn is a Python module integrating a wide range of state-of-the-art ML algorithms for medium-scale supervised and unsupervised problems [23]. The LR classifier, the random forest classifier (RFC), and the gradient boosting classifier (GBC) have been typically used to construct classifiers in prediction of disease risk, progression, prognosis, and so on [24]. The LR classifier in the *sklearn.linear_model* is also known as logit regression, maximum-entropy classification, or the log-linear classifier. In this model, the probabilities describing the possible outcomes of a single trial are modeled using a logistic function [24]. The RFC in the *sklearn.ensemble* module is one of the averaging algorithms in ensemble methods and is a perturb-and-combine technique specifically designed for trees. In the random forest algorithm, each tree in the ensemble is built from a sample drawn with replacement from the training data set. Furthermore, when splitting each node during the construction of a tree, the best split is found either from all input features or from a random subset of size setting with the parameter *max_features*. In practice, the variance reduction due to the introduction of randomness in the classifier construction is often significant, hence, yielding an overall better model [25,26]. The GBC algorithm, using the *sklearn.ensemble* function, is a boosting method, in which base estimators are built sequentially. To reduce the bias of the combined estimator, one has to combine several weak models to produce a powerful ensemble. The GBC algorithm builds an additive model in a forward stage-wise fashion, and it allows for the optimization of arbitrary differentiable loss functions [27,28].

In this study, the classifiers were respectively constructed using the LR classifier, RFC, and GBC in the scikit-learn module with the training data set. The model parameter settings were kept as default, except that *random_state* was modified to “0” for all models and *class_weight* was modified to “balanced” for the LR classifier and RFC models. The performance of the classifiers was evaluated with the test data set by calculating the recall rate (ie, sensitivity), specificity, accuracy, and area under the receiver operating characteristic curve (AUROC), using the *sklearn_metrics.recall_score*, *sklearn_metrics.precision_score*, *sklearn_metrics.accuracy_score*, and *sklearn_metrics.auc* functions, respectively. Gini importance was computed using the *feature_importance* function to measure the importance of each feature in the RFC and the GBC. The higher the Gini importance value, the more important the feature [29]. All the above analyses were performed in Python, version 3.7 (Python Software Foundation).

## Results

### Basic Characteristics of CAP Group and COVID-19 Group

No significant differences in age and sex were found between CAP and COVID-19 groups (see Table 1); however, the proportions of males in the CAP and COVID-19 groups were 55% (33/60) and 66% (40/61), respectively, and were higher than those of females in both groups. No significant difference in the medical history between the two groups (see Table 1) was observed. Also, no significant difference was found in the proportions of the main clinical symptoms between the two groups, such as fever, cough, fatigue, muscle soreness, and loss of appetite (see Table 1). The average hospitalization days for CAP patients were remarkably lower than those for COVID-19 patients (*P*<.001). In the CAP group, some patients with pulmonary CT also had imaging features that included patchy hyperdense shadow (11/60, 18%), ground-glass shadow (4/60, 7%), and fibrotic lesion (6/60, 10%). Nonetheless, the chief imaging features of pulmonary CT in the COVID-19 group were patchy hyperdense shadow (25/61, 41%) and ground-glass shadow (9/61, 15%), and many patients (7/61, 11%) had both patchy hyperdense shadow and ground-glass shadow (see Table 1). Among the 61 patients suffering from COVID-19, 3 (5%) had mild symptoms, 47 (77%) had common symptoms, 6 (10%) had severe symptoms, and 5 (8%) had critical symptoms. Fever and cough were the principal symptoms in the early stage of COVID-19, and these accounted for 70% (43/61) and 64% (39/61) of the cases, respectively (see Table 1). Among the CAP patients included in the analysis, no cases of death were found during hospitalization; however, 3 of the 5 (60%) severely ill patients in the COVID-19 group, who were aged 36, 49, and 74 years, died during hospitalization. The 36-year-old patient who died underwent interventricular septal repair in childhood.

**Table 1:** Comparison of baseline information between COVID-19 patients and community-acquired pneumonia (CAP) patients.

Baseline characteristic		CAP patients (n=60)	COVID-19 patients (n=61)	*P* value
Sex (male), n (%)		33 (55)	40 (66)	.27
Age (years), mean (SD)		55.72 (18.10)	50.23 (16.95)	.09
Hospitalization days, median (IQR)		9 (7-12)	21 (13-26)	<.001
**Medical history, n (%)**				
	Hypertension	14 (23)	16 (26)	.83
	Diabetes	2 (3)	6 (10)	.27
	Liver disease	2 (3)	3 (5)	.99
	Heart disease	3 (5)	5 (8)	.72
	Exposure history	Unclear	54 (89)	N/A^a^
	Familial aggregation infection^b^	Unclear	22 (36)	N/A
**Initial symptoms, n (%)**				
	Fever	36 (60)	43 (70 )	.26
	Cough	44 (73)	39 (64)	.33
	Myalgia	4 (7)	7 (11)	.53
	Poor appetite	5 (8)	11 (18)	.18
	Fatigue	33 (55)	24 (39)	.10
Days from onset of symptoms to admission, median (IQR)		Unrecorded	3 (1-7)	N/A
**Imaging features, n (%)**				
	Patchy high-density opacity	11 (18)	25 (41)	.009
	Ground-glass opacity	4 (7)	9 (15)	.24
	Fibrotic lesion	6 (10)	3 (5)	.32
	Patchy high-density opacity and ground-glass opacity	0 (0)	7 (11)	.01
Death cases, n (%)		0 (0)	3 (5)	N/A

^a^N/A: not applicable; groups could not be compared because there were no values for the CAP group.

^b^There were more than 2 cases of infection after aggregation with family members or relatives.

### Characteristic Profile of the CLIs in COVID-19 and CAP

**Figure 1:**
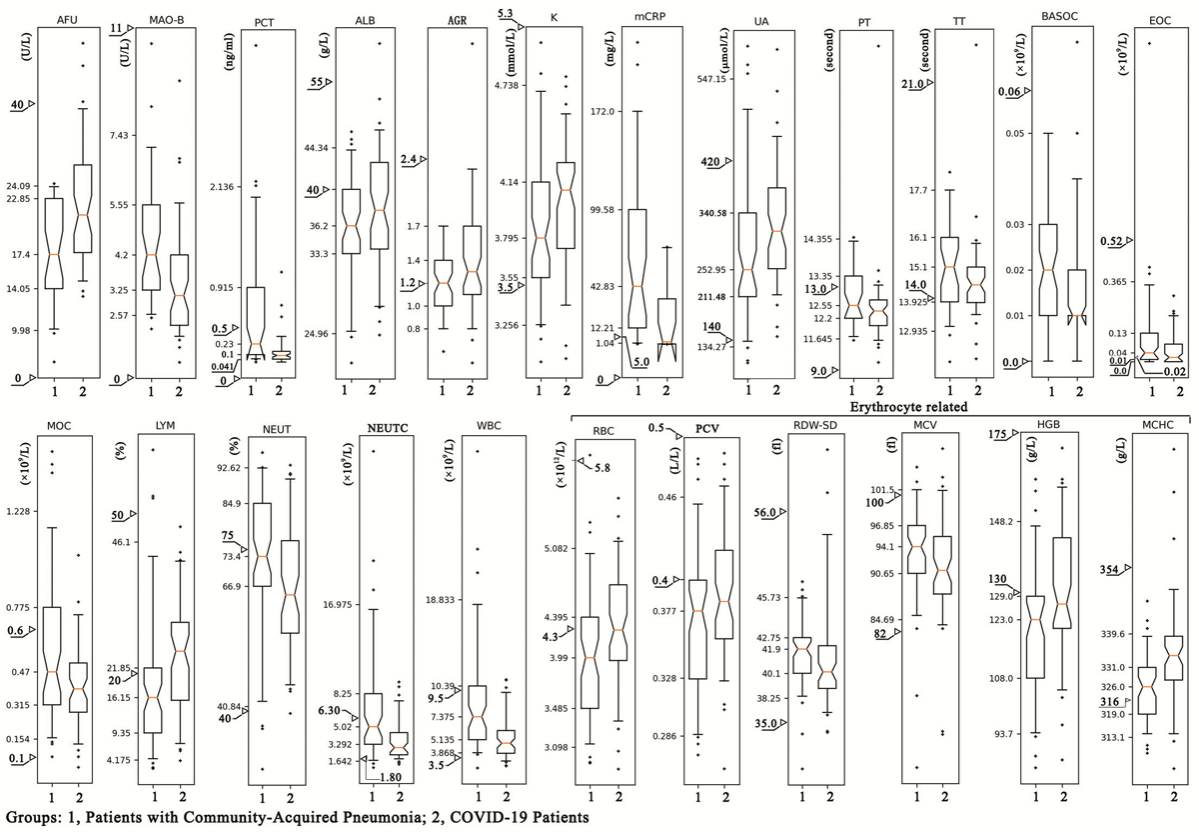
The statistical distribution of the plasma level of the clinical laboratory indicators (CLIs) with a remarkable difference between COVID-19 and community-acquired pneumonia (CAP). The statistical distribution was presented with a box and whisker plot. The horizontal lines within the boxes indicate the median value. The vertical lines extending below and above the boxes represent 5%-95% percentile values. The scale on the y-axis represents the values of the 5th, 25th, 50th, 75th, and 95th percentiles of the CLI in the CAP group. The triangles represent the upper and lower limits of the normal reference range of the laboratory index. AFU: α-L-fucosidase; AGR: albumin to globulin ratio; ALB: albumin; BASOC: basophil count; EOC: eosinophil count; HGB: hemoglobin concentration; K: potassium; LYM: lymphocyte; MAO-B: monoaminoxidase B; MCHC: mean corpuscular hemoglobin concentration; mCRP: micro–C-reactive protein; MCV: mean (red blood cell) corpuscular volume; MOC: monocyte count; NEUT: neutrophil ratio; NEUTC: neutrophil count; PCT: procalcitonin; PCV: packed-cell volume (hematocrit); PT: prothrombin time; RBC: red blood cell count; RDW-SD: red blood cell distribution width–standard deviation; TT: thrombin time; UA: uric acid; WBC: white blood cell count.

Even though most CLIs had a similar variation trend in both CAP and COVID-19, the extent of change was different. Among more than 60 evaluated CLIs, there were significant differences in 25 CLIs between the two groups (see Table 2). A decrease of lymphocyte, red blood cell (RBC) count, hematocrit or packed-cell volume (PCV), hemoglobin concentration, and mean corpuscular hemoglobin concentration (MCHC) and an increase of neutrophil ratio, prothrombin time (PT), micro-CRP (mCRP), and PCT were observed in both COVID-19 and CAP patients. Furthermore, the neutrophil ratio and levels of PT, mCRP, and PCT in CAP were remarkably higher than those in COVID-19. Levels of lymphocyte, RBC count, PCV, hemoglobin concentration, and MCHC in CAP were significantly lower than those in COVID-19 (see Figure 1). Various erythrocyte-related CLIs—RBC count, PCV, hemoglobin concentration, and MCHC—significantly decreased in both CAP and COVID-19, but there was a greater reduction in CAP patients (see Figure 1). The RBC distribution width–standard deviation (RDW-SD) and RBC mean corpuscular volume (MCV) also indicated prominent differences between CAP and COVID-19 (see Figure 1).

**Table 2:** Differences in clinical laboratory indicators (CLIs) between patients with community-acquired pneumonia (CAP) and COVID-19.

CLI	CAP patients (n=60)		COVID-19 patients (n=61)		*P* value
	n (%)	Mean (SD)	n (%)	Mean (SD)	
Procalcitonin (ng/mL)	43 (72)	0.629 (0.838)	55 (90)	0.134 (0.184)	<.001
Monoamine oxidase B (U/L)	35 (58)	4.569 (1.748)	53 (87)	3.538 (1.592)	.001
Myoglobin (ng/mL)	14 (23)	39.179 (29.421)	23 (38)	65.794 (87.039)	.04
Micro–C-reactive protein (mg/L)	41 (68)	63.943 (64.530)	13 (21)	22.568 (29.577)	.004
Prothrombin time (seconds)	30 (50)	12.780 (0.873)	53 (87)	12.460 (1.107)	.04
Thrombin time (seconds)	30 (50)	15.123 (1.565)	53 (87)	14.655 (1.422)	.049
Albumin (g/L)	53 (88)	35.508 (5.929)	54 (89)	37.831 (6.169)	.04
Albumin to globulin ratio	53 (88)	1.211 (0.295)	54 (89)	1.378 (0.482)	.047
α-L-fucosidase (U/L)	35 (58)	17.709 (5.167)	50 (82)	22.106 (5.698)	<.001
Uric acid (μmol/L)	44 (73)	284.193 (118.608)	54 (89)	325.261 (92.914)	.007
Potassium (mmol/L)	54 (90)	3.900 (0.462)	55 (90)	4.021 (0.392)	.03
White blood count cell (×10^9^/L)	58 (97)	8.858 (5.576)	56 (92)	5.293 (2.047)	<.001
Neutrophils (%)	57 (95)	72.958 (15.544)	56 (92)	66.661 (14.013)	.007
Lymphocytes (%)	56 (93)	18.646 (13.416)	56 (92)	24.014 (11.175)	.002
Neutrophil count (×10^9^/L)	56 (93)	6.797 (5.525)	56 (92)	3.649 (1.949)	<.001
Monocyte count (×10^9^/L)	55 (92)	0.565 (0.337)	56 (92)	0.404 (0.194)	.009
Eosinophil count (×10^9^/L)	55 (92)	0.111 (0.213)	56 (92)	0.053 (0.072)	.03
Basophil count (×10^9^/L)	55 (92)	0.021 (0.013)	56 (92)	0.015 (0.013)	.002
Red blood cell count (×10^12^/L)	56 (93)	4.028 (0.647)	56 (92)	4.284 (0.570)	.008
Hemoglobin concentration (g/L)	55 (92)	120.800 (17.326)	56 (92)	130.143 (16.888)	.005
Packed-cell volume (hematocrit) (L/L)	55 (92)	0.371 (0.052)	56 (92)	0.389 (0.049)	.04
Mean red blood cell volume (fL)	55 (92)	93.255 (6.662)	56 (92)	91.241 (6.501)	.01
Mean corpuscular hemoglobin concentration (g/L)	55 (92)	325.473 (8.360)	56 (92)	334.482 (13.559)	<.001
Red blood cell distribution width–standard deviation (fL)	55 (92)	41.476 (2.573)	56 (92)	41.141 (4.082)	.01

Comparing the COVID19-COM and COVID19-SV subgroups, 26 CLIs demonstrated a remarkable difference (see Table 3). In comparison with the COVID19-COM subgroup, LDH, aspartate aminotransferase, fibrinogen content, mCRP, and erythrocyte sedimentation rate increased acutely in the COVID19-SV subgroup, whereas prealbumin, carbon dioxide binding capacity, lymphocytes, and lymphocyte count decreased in the COVID19-SV subgroup (see Multimedia Appendix 1).

An orderly increase of α-L-fucosidase (AFU), myoglobin, uric acid, and MCHC and an orderly decrease of thrombin time, monocyte count, eosinophil count, RBC MCV, and RDW-SD were observed in CAP, COVID19-COM, and COVID19-SV patients, indicating that these CLIs may be used to distinguish CAP from COVID-19 and may suggest the probability of severe COVID-19 progression (see Multimedia Appendix 2).

**Table 3:** Difference in clinical laboratory indicators (CLIs) between patients with common and severe types of COVID-19.

CLIs	Patients with a common type of COVID-19 (n=50)		Patients with a severe type of COVID-19 (n=11)		*P* value
	n (%)	Mean (SD)	n (%)	Mean (SD)	
Procalcitonin (ng/mL)	44 (88)	0.112 (0.170)	11 (100)	0.224 (0.217)	.01
N-terminal pro-B-type natriuretic peptide (pg/mL)	29 (58)	366.053 (549.429)	11 (100)	534.782 (398.067)	.03
Hypersensitive C-reactive protein (mg/L)	41 (82)	23.332 (34.483)	11 (100)	72.458 (60.805)	.002
Lactate dehydrogenase (U/L)	26 (52)	214.896 (73.319)	8 (73)	314.750 (118.755)	.02
D-dimer (mg/L)	42 (84)	0.834 (1.115)	11 (100)	5.133 (10.399)	.005
Myoglobin (ng/mL)	16 (32)	49.221 (60.505)	7 (64)	103.674 (127.354)	.02
Cardiac troponin (ng/mL)	16 (32)	0.011 (0.003)	7 (64)	0.033 (0.041)	.02
Creatine kinase (U/L)	27 (54)	81.296 (47.153)	8 (73)	202.125 (195.052)	.02
Fibrinogen content (mg/dL)	42 (84)	411.905 (104.363)	11 (100)	467.455 (76.500)	.03
Aspartate aminotransferase (U/L)	46 (92)	29.413 (15.756)	10 (91)	45.600 (18.969)	.004
γ-glutamyl transpeptidase (U/L)	44 (88)	46.046 (41.609)	10 (91)	80.000 (44.229)	.007
Albumin (g/L)	44 (88)	38.602 (6.267)	10 (91)	34.440 (4.558)	.02
Albumin to globulin ratio	44 (88)	1.436 (0.507)	10 (91)	1.120 (0.230)	.02
Indirect bilirubin (μmol/L)	44 (88)	9.482 (3.841)	10 (91)	7.960 (4.336)	.048
Prealbumin (mg/L)	41 (82)	180.171 (83.374)	9 (82)	125.556 (68.182)	.03
β2-microglobulin (mg/L)	41 (82)	1.978 (0.430)	9 (82)	2.528 (1.015)	.01
Carbon dioxide binding capacity (mmol/L)	41 (82)	25.420 (2.537)	9 (82)	22.733 (2.018)	.002
Potassium (mmol/L)	44 (88)	4.057 (0.414)	11 (100)	3.876 (0.251)	.04
Erythrocyte sedimentation rate (mm/h)	30 (60)	55.433 (41.639)	7 (64)	87.000 (35.081)	.02
Neutrophils (%)	45 (90)	64.496 (13.286)	11 (100)	75.519 (14.001)	.02
Lymphocytes (%)	45 (90)	25.711 (10.932)	11 (100)	17.073 (9.750)	.01
Eosinophils (%)	45 (90)	1.236 (1.388)	11 (100)	0.391 (1.038)	.009
Eosinophil count (×10^9^/L)	45 (90)	0.062 (0.076)	11 (100)	0.014 (0.039)	.003
Lymphocyte count (×10^9^/L)	45 (90)	1.255 (0.558)	11 (100)	0.835 (0.383)	.008
Packed-cell volume (hematocrit) (L/L)	45 (90)	0.395 (0.050)	11 (100)	0.368 (0.036)	.03
Red blood cell distribution width–coefficient of variation (%)	45 (90)	12.658 (1.171)	11 (100)	12.873 (0.781)	.03

### Classifiers Constructed From the FCs With Seven to Eight CLIs Could Accurately Distinguish COVID-19 From CAP

**Figure 2:**
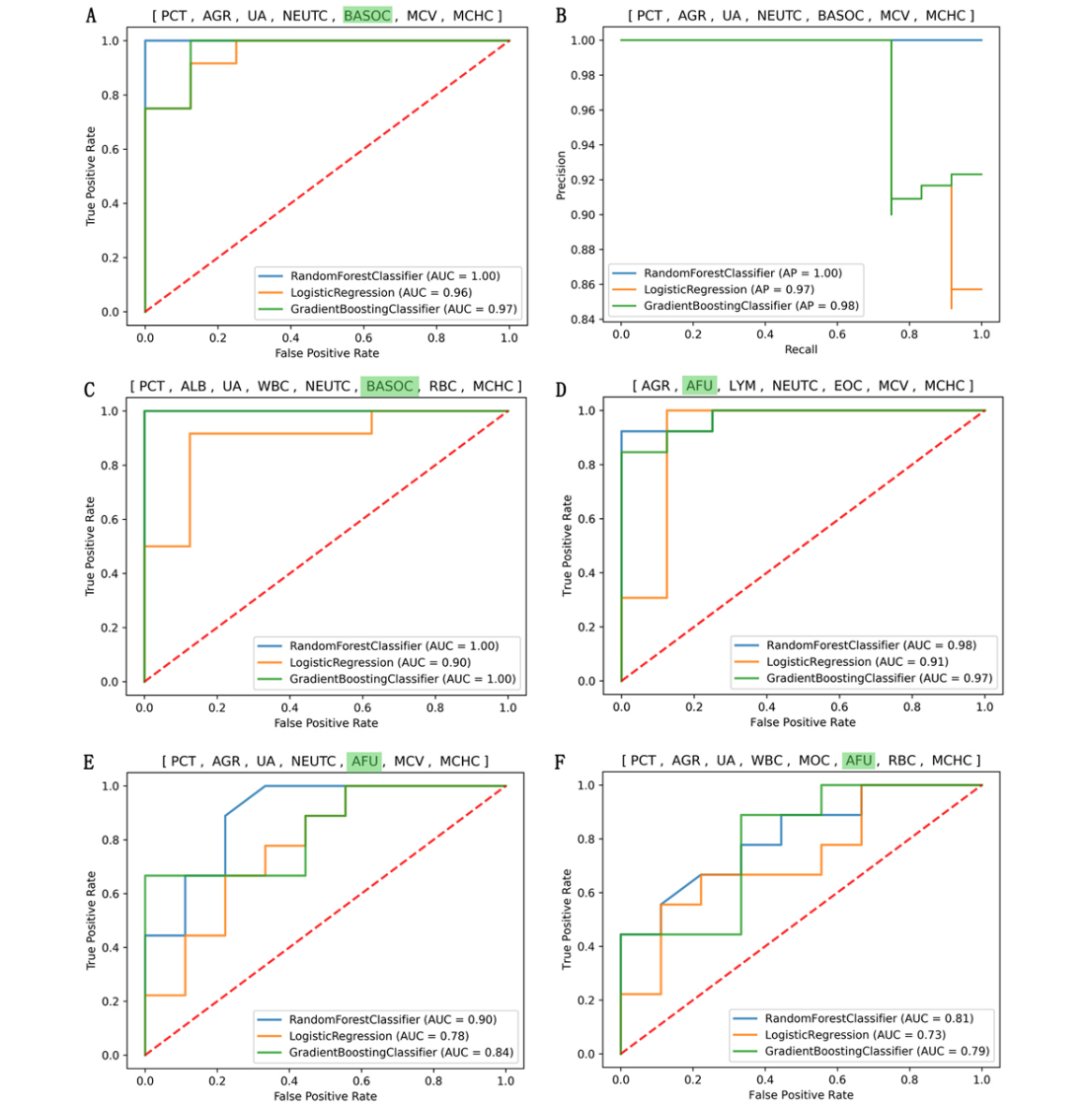
Area under the receiver operating characteristic curve (AUROC) and precision-recall curve plotted for the COVID-19 vs community-acquired pneumonia (CAP) classifiers built with various feature combinations (FCs) of different clinical laboratory indicators (CLIs). At the top of each image is the CLI combination for constructing classifiers using three different classification algorithms. AFU: α-L-fucosidase; AGR: albumin to globulin ratio; ALB: albumin; BASOC: basophil count; EOC: eosinophil count; LYM: lymphocyte; MCHC: mean corpuscular hemoglobin concentration; MCV: mean (red blood cell) corpuscular volume; MOC: monocyte count; NEUTC: neutrophil count; PCT: procalcitonin; RBC: red blood cell count; UA: uric acid; WBC: white blood cell count.

**Figure 3:**
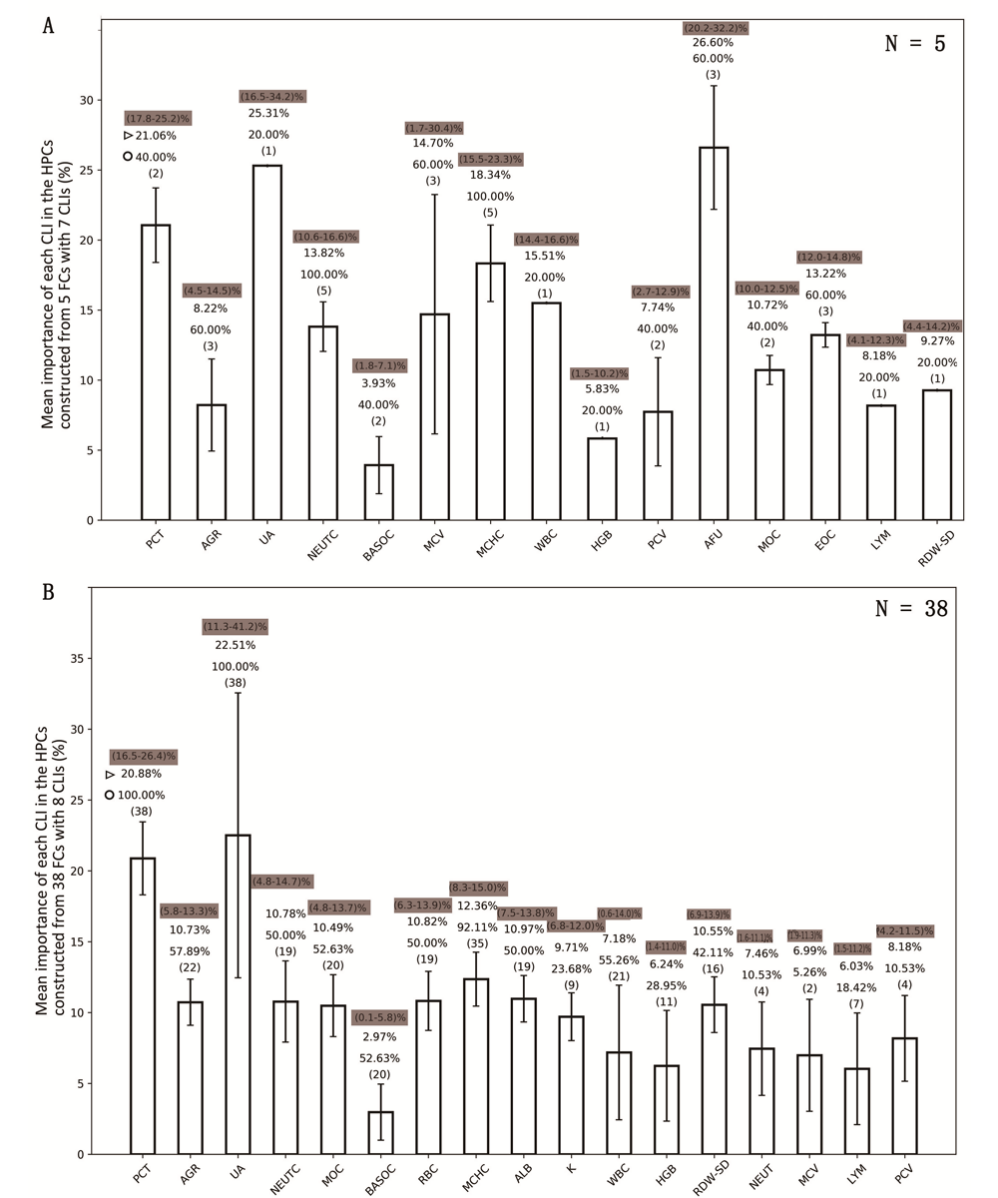
Usage rate and the feature importance of each clinical laboratory indicator (CLI) in the high-performance COVID-19 vs community-acquired pneumonia (CAP) classifiers. (A) The mean feature importance of each CLI in the high-performance classifiers (HPCs) constructed with the 7-CLI combinations. (B) The mean feature importance of each CLI in the HPCs constructed with the 8-CLI combinations. The histogram is represented by mean (SD). The numbers with the shadow backgrounds represent the minimum and maximum values of the feature importance of the CLI. The number indicated with the triangle symbol represents the mean feature importance of CLI in all classifiers. The number indicated with the circle represents the usage rate of the CLI in the HPC. The number in the parentheses indicates how many CLI combinations are capable of constructing the HPCs containing the CLI. AFU: α-L-fucosidase; AGR: albumin to globulin ratio; ALB: albumin; BASOC: basophil count; EOC: eosinophil count; FC: feature combination; HGB: hemoglobin concentration; K: potassium; LYM: lymphocyte; MCHC: mean corpuscular hemoglobin concentration; MCV: mean (red blood cell) corpuscular volume; MOC: monocyte count; NEUT: neutrophil ratio; NEUTC: neutrophil count; PCT: procalcitonin; PCV: packed-cell volume (hematocrit); RBC: red blood cell count; RDW-SD: red blood cell distribution width–standard deviation; UA: uric acid; WBC: white blood cell count.

The performance of the classifiers gradually improved as the number of CLIs in the FCs increased from one to eight. However, when the number of CLIs in the FCs reached eight, the performance of the classifiers constructed by these FCs no longer significantly improved. The performance of the LR classifier algorithm constructed with the FCs with eight CLIs (ie, 8-CLI combination) was even slightly lower than those constructed by the FCs with seven CLIs (ie, 7-CLI combination). A total of 43 FCs, including five 7-CLI combinations and 38 8-CLI combinations, were determined according to the recall rate. The AUROCs of the classifiers constructed with the LR classifier, RFC, and GBC algorithms were greater than 0.85 (see Multimedia Appendix 3, Table S1). The AUROC and precision-recall curves of the classifiers constructed with the RFC, LR classifier, and GBC algorithms from the representative 7-CLI combination (ie, PCT, albumin to globulin ratio [AGR], uric acid, neutrophil count, basophil count, RBC MCV, and MCHC) showed very high performance and precision in COVID-19 prediction; their AUROCs were 1.0, 0.97, and 0.96, respectively (see Figure 2, A), and their average precision values were 1.0, 0.97, and 0.98, respectively (Figure 2, B). The AUROCs of the classifiers constructed with the RFC, LR classifier, and GBC algorithms from the representative 8-CLI combination (ie, PCT, albumin, uric acid, WBC [white blood cell] count, monocyte count, basophil count, RBC count, and MCHC) were 1.0, 0.90, and 1.0, respectively (see Figure 2, C). The AUROCs of the classifiers constructed with the three algorithms from the 7-CLI combination (ie, agr, afu, lymphocytes, neutrophil counts, eosinophil count, RBC mcv, and mchc) were 0.98, 0.91, and 0.97, respectively (see Figure 2, D). Feature importance results showed that basophil count was the least important in the above two representative CLI combinations, and AFU was the most important in the CLI combinations (see Figure 3). However, when basophil count was substituted with AFU in the two above-mentioned CLI combinations, the performance of the classifiers constructed with the new CLI combinations decreased (see Figure 2, E and F). PCT and AFU were not observed to be in the same CLI combination from which an HPC could be constructed. The evidence above and the fact that only 43 FCs with seven or eight CLIs could be used to build HPCs suggested that only the FCs with specific CLIs can establish HPCs to distinguish COVID-19 from CAP.

The importance of different CLIs in classifiers varied greatly, and the importance of the same CLI varied greatly among classifiers constructed by different FCs (see Figure 3). In the HPCs constructed with the 7-CLI combinations, the average feature importance of AFU (26.60%) was the highest, followed by uric acid (25.31%) and PCT (21.06%) (see Figure 3, A). However, in the HPCs constructed with the 8-CLI combinations, the average feature importance of uric acid (22.51%) was the highest, followed by PCT (20.88%) and MCHC (12.36%) (see Figure 3, B). PCT and MCHC were very important to each classifier because they were included, respectively, in 100% (38/38) and 92% (35/38) of the 8-CLI combinations (see Figure 3, B) and in 40% (2/5) and 100% (5/5) of the 7-CLI combinations (see Figure 3, A). Uric acid was also included in all 8-CLI combinations, but its feature importance varied from 11.3% to 41.2% in different classifiers (see Figure 3, B).

## Discussion

### Principal Findings

The main highlight of this study is that only a few of the common CLIs were required to establish the classifier models to accurately distinguish COVID-19 from CAP. The HPCs could only be constructed by combining several specific CLIs. Among the nearly 2 million FCs with one to eight CLIs, only 43 FCs could be used to construct HPCs with a recall rate greater than 0.9 and an AUROC greater than 0.85 to distinguish COVID-19 from CAP.

### Comparison With Prior Work

We have established many COVID-19 versus CAP HPCs with FCs consisting of only CLIs, and almost no similar research results on distinguishing COVID-19 from CAP have been reported. However, many studies have used CLIs to build ML models to help with COVID-19 diagnosis. The prediction performance of these models varied: the accuracy of these models in predicting COVID-19 was between 0.8 and 0.96 [30-32]. In addition, most of the reported ML models for the diagnosis or prediction of COVID-19 have involved more types of variables, such as CT results, clinical symptoms, and CLIs [17,32,33]. Although most of these COVID-19-related ML models were built with more than two ML algorithms, not all models constructed with each algorithm showed high performance. The methods of feature selection that were used in these studies included the recursive feature elimination algorithm [31], causal explanation models [17], and the least absolute shrinkage and selection operator regression [32]. These methods can extract the features that are closely related to the target phenotype, but whether the classifier constructed by the combination of these features has the best performance needs to be determined. The optimized FCs in this study were selected by evaluating the recall rate and AUROC for each FC with one to eight randomly selected CLIs from the differential CLIs between COVID-19 and CAP groups and by constructing classifiers using each FC with the LR classifier algorithm. The FCs that were preliminarily screened were used to build classifiers with RFC and GBC algorithms; finally, only the FCs capable of building the HPC simultaneously with the LR classifier, RFC, and GBC algorithms were selected for the final model construction.

### Limitations

As reported earlier, many inflammatory factors, including IL-6 and interleukin-10 (IL-10), are closely related to COVID-19 and have diagnostic value, but neither IL-6 nor IL-10 were detected in the patients of this study. Menni et al [18] reported that loss of smell and taste is a strong predictor for COVID-19. Deviations and omissions may exist in the patients’ self-reported clinical symptoms. Thus, we did not take into account the clinical symptoms when building the classifiers. The possibility that other indicators are more important in constructing COVID-19 versus CAP classifiers was not ruled out. In addition, the sample size included in this study was relatively small, and the classifiers need to be optimized with larger samples before it can be used to distinguish COVID-19 from CAP in practice.

### The Rationality of the Research Results

Out of the 43 FCs, 40 contained PCT and MCHC. The feature importance of PCT in each classifier is very high, suggesting that PCT may be a good blood marker to efficiently distinguish COVID-19 from CAP. PCT is one of the markers of lower respiratory tract bacteria and other infections. The US Food and Drug Administration approved the monitoring of the beginning and the entire duration of antibiotic treatment for suspected lower respiratory tract infections based on serum PCT levels [12]. However, the elevation of serum PCT in COVID-19 patients was also reported in many studies [34]. The increase of PCT is a remarkable characteristic of patients with COVID-19 [34]. Increased serum PCT levels in both COVID-19 and CAP patients indicated that the distinction of COVID-19 from CAP could not be made simply on the basis of the increase in PCT. Compared with the normal reference values of the CLIs, the serum levels of most of the CLIs increased or decreased simultaneously in both COVID-19 and CAP patients. Thus, providing references for the diagnosis of COVID-19 or CAP directly in regard to the rise or decrease of the CLIs is difficult. However, we found that the ML classifiers constructed with the FCs with many certain CLIs could distinguish COVID-19 from CAP effectively, suggesting an advantage of ML algorithms in disease classification or diagnosis.

The COVID-19 versus CAP classifiers with the highest performance also involved PCT, MCHC, uric acid, albumin, neutrophil count, monocyte count, basophil count, RBC count, and WBC count, proposing the importance of these CLIs in differentiating COVID-19 from CAP. Few studies have reported the changing trend of MCHC in patients with COVID-19 or CAP, but the results of this study showed that MCHC decreased in both groups and was significantly lower in the CAP group than in the COVID-19 group. The reason for the decrease of MCHC may be closely related to the reduction of iron due to inflammation [35]. The IQRs of uric acid in both COVID-19 and CAP groups were within the normal reference range, but the IQR was significantly higher in the COVID-19 group than in the CAP group. Elevated uric acid is an independent risk factor of renal injury or renal dysfunction; the underlying mechanisms of uric acid elevation are very complicated [36]. The significant difference in uric acid between COVID-19 and CAP may be interpreted as follows: individuals with higher uric acid may be more susceptible to COVID-19 than those with lower uric acid levels. Uric acid exists in all 8-CLI combinations that are capable of constructing high-performance CLIs and has a high feature importance in the classifiers, suggesting that uric acid is another important marker that can distinguish COVID-19 from CAP. Zhou et al reported that albumin significantly decreased in severe and critical COVID-19 patients [37]. Serum albumin level is a good prognostic marker in CAP. A decreased albumin level is closely associated with a higher risk of mortality in patients with CAP [38]. Although albumin decreased remarkably in both COVID-19 and CAP groups, there was still a significant difference between the two groups; the decrease in the CAP group was more obvious than that in the COVID-19 group, which could contribute to the differentiation of COVID-19 from CAP. AFU contributed high feature importance in the HPCs constructed from 7-CLI combinations due to the significant difference in AFU between COVID-19 and CAP. An increase of serum AFU has a certain diagnostic value for primary liver cancer [39]. Thus, the higher AFU in the COVID-19 group than in the CAP group may be explained by the fact that liver injury is more common in COVID-19 than in CAP or that the diversity in AFU levels determines the difference in susceptibility to COVID-19.

### Recommendations

Both PCT and AFU contributed high feature importance in the HPCs constructed from the FCs containing PCT or AFU, but the performance of the classifiers constructed from the FCs containing both PCT and AFU decreased remarkably. This result indicated that intrinsic dependence exists among some CLIs that undergo synergistic changes in individuals and can be used to construct HPCs. The internal relationship between CLIs is very complex and difficult to deconstruct. Therefore, the following method may be effective: random selection of different CLIs to construct classifiers with different classification algorithms, followed by the evaluation of the performance of each classifier, and, finally, the discovery of the FCs with certain CLIs that can be used to accurately distinguish COVID-19 from CAP.

### Conclusions

The patients suffering from COVID-19 and CAP have their own characteristic profiles of CLIs, and some FCs consisting of seven or eight specific CLIs could build COVID-19 versus CAP HPCs. The usage rate and the feature importance of the CLIs in the HPCs indicated that PCT, MCHC, uric acid, albumin, AGR, neutrophil count, RBC count, monocyte count, and WBC count are the most important indicators that can distinguish COVID-19 from CAP.

## Appendix

Multimedia Appendix 1 The statistical distribution of the plasma level of the clinical laboratory indicators (CLIs) with a significant difference between COVID19-COM (COVID-19 patient subgroup with mild and common types) and COVID19-SV (COVID-19 patient subgroup with severe and critical types). The statistical distribution was presented with a box and whisker plot. The horizontal lines within the boxes indicate the median value. The vertical lines extending below and above the boxes represent 5%-95% percentile values. The scale on the y-axis represents the 5th, 25th, 50th, 75th, and 95th percentile values of the CLI in the COVID19-COM subgroup. The triangles represent the upper and lower limits of the normal reference range of the laboratory index. The median of the CLI in the COVID19-SV subgroup is also represented in the y-axis. AST: aspartate aminotransferase; CO2CP: carbon dioxide binding capacity; ESR: erythrocyte sedimentation rate; γ-GGT: transglutaminase transpeptidase gamma; FIB: fibrinogen content; LDH: lactate dehydrogenase; LYM: lymphocyte; LYMPH: lymphocyte count; mCRP: micro–C-reactive protein; MYO: myoglobin; NEUT: neutrophil ratio; PA: prealbumin.

Multimedia Appendix 2 The statistical distribution of the plasma level of the clinical laboratory indicators (CLIs) among community-acquired pneumonia (CAP), COVID19-COM (COVID-19 patient subgroup with mild and common types), and COVID19-SV (COVID-19 patient subgroup with severe and critical types). The statistical distribution was presented with a box and whisker plot. The horizontal lines within the boxes indicate the median value. The vertical lines extending below and above the boxes represent 5%-95% percentile values. The scale on the y-axis represents the 5th, 25th, 50th, 75th, and 95th percentile values of the CLI in the CAP group. The triangles represent the upper and lower limits of the normal reference range of the laboratory index. AFU: α-L-fucosidase; EOC: eosinophil count; MCHC: mean corpuscular hemoglobin concentration; MCV: mean (red blood cell) corpuscular volume; MOC: monocyte count; MYO: myoglobin; RDW-SD: red blood cell distribution width–standard deviation; TT: thrombin time; UA: uric acid.

Multimedia Appendix 3 Clinical laboratory indicator (CLI) combinations and the hyper-parameters of the classifiers constructed by different machine learning algorithms from these CLI combinations.

## Abbreviations

AFU: α-L-fucosidase
AGR: albumin to globulin ratio
AUROC: area under the receiver operating characteristic curve
CAP: community-acquired pneumonia
CLI: clinical laboratory indicator
COVID19-COM: COVID-19 patient subgroup with mild and common types
COVID19-SV: COVID-19 patient subgroup with severe and critical types
CRP: C-reactive protein
CT: computed tomography
FC: feature combination
GBC: gradient boosting classifier
HPC: high-performance classifier
IL-6: interleukin-6
IL-10: interleukin-10
LDH: lactate dehydrogenase
LR: logistic regression
MCHC: mean corpuscular hemoglobin concentration
mCRP: micro–C-reactive protein
MCV: mean corpuscular volume
ML: machine learning
PCT: procalcitonin
PCV: packed-cell volume
PT: prothrombin time
RBC: red blood cell
RDW-SD: red blood cell distribution width–standard deviation
RFC: random forest classifier
WBC: white blood cell
