# Supplementary figures and images for: Establishing Classifiers With Clinical Laboratory Indicators to Distinguish COVID-19 From Community-Acquired Pneumonia: Retrospective Cohort Study
Source: J Med Internet Res. 2021 Feb 22;23(2):e23390. doi: 10.2196/23390 (PMC7901596; doi:10.2196/23390)

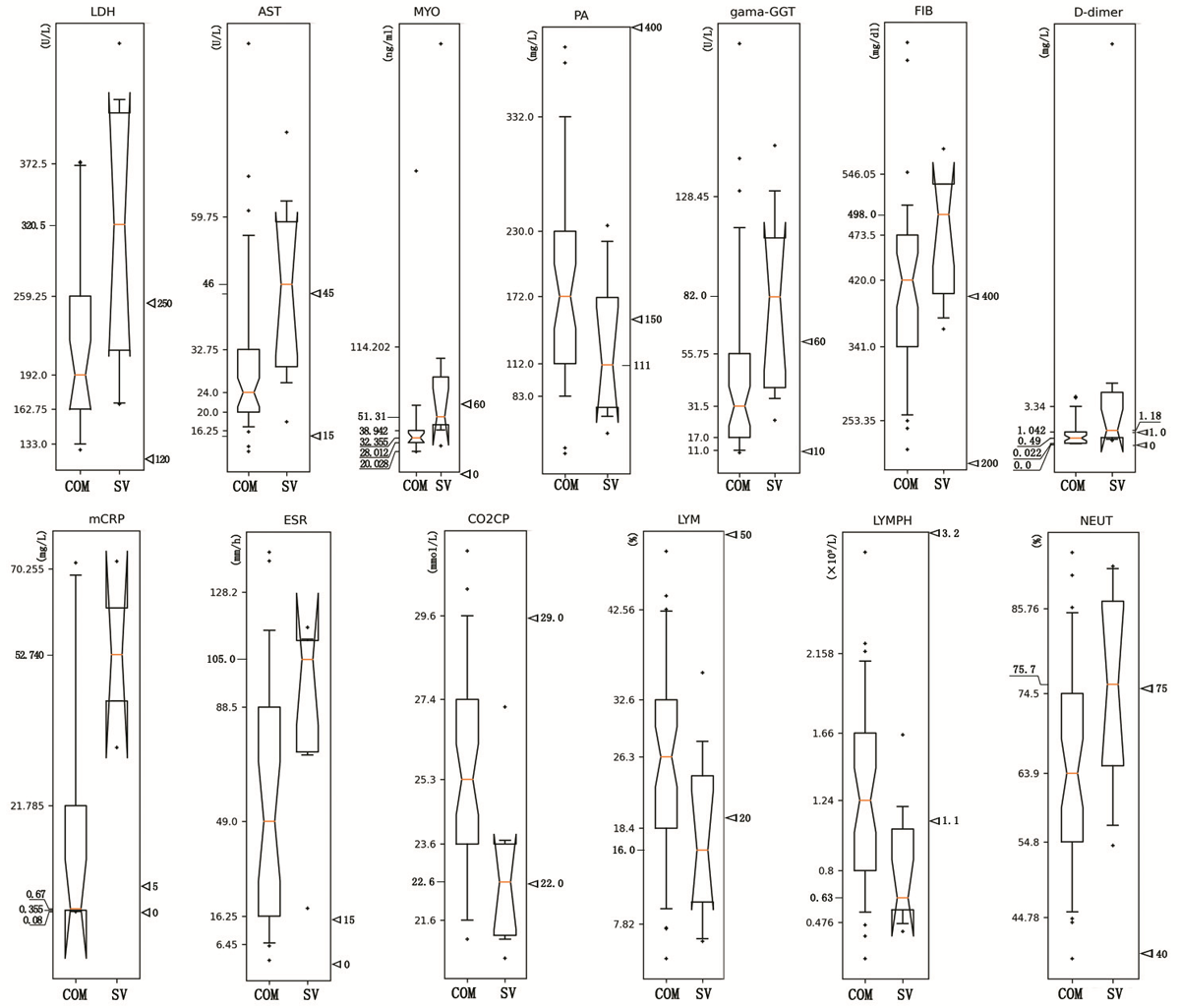

Supplement: Multimedia Appendix 1 [file jmir_v23i2e23390_app1.png]

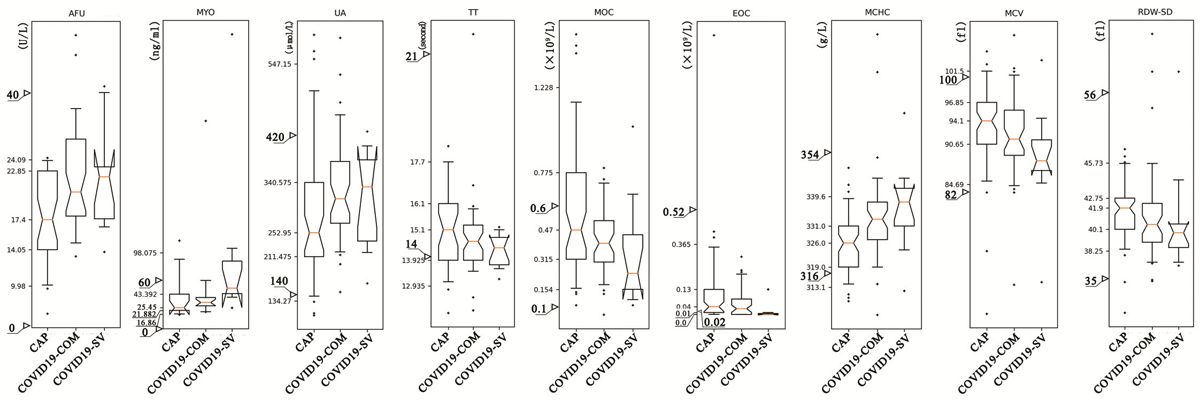

Supplement: Multimedia Appendix 2 [file jmir_v23i2e23390_app2.png]
